# A new animal model of atrophy–hypertrophy complex and liver damage following Yttrium-90 lobar selective internal radiation therapy in rabbits

**DOI:** 10.1038/s41598-022-05672-3

**Published:** 2022-02-02

**Authors:** María Páramo, Eva Santamaría, Miguel A. Idoate, Macarena Rodríguez-Fraile, Alberto Benito, Maria Collantes, Gemma Quincoces, Iván Peñuelas, Carmen Berasain, Josepmaria Argemi, Jorge Quiroga, Bruno Sangro, José I. Bilbao, Mercedes Iñarrairaegui

**Affiliations:** 1 Department of Radiology, Clínica Universidad de Navarra, Pamplona, Spain; 2 Hepatology Program, Center for Applied Medical Research (CIMA), Universidad de Navarra, Pamplona, Spain; 3 CIBERehd, Instituto de Salud Carlos III, Madrid, Spain; 4 Department of Pathology, Clínica Universidad de Navarra, Pamplona, Spain; 5 Department of Nuclear Medicine, Clínica Universidad de Navarra, Pamplona, Spain; 6 Instituto de Investigaciones Sanitarias de Navarra-IdiSNA, Pamplona, Spain; 7 Radiopharmacy, Radionanopharmacology and Translational Molecular Imaging Research Group, Clínica Universidad de Navarra, Pamplona, Spain; 8 Radiopharmacy Unit, Department of Nuclear Medicine, Clínica Universidad de Navarra, Pamplona, Spain; 9 Liver Unit, Clínica Universidad de Navarra, Pamplona, Spain

**Keywords:** Translational research, Experimental models of disease, Liver

## Abstract

Lobar selective internal radiation therapy (SIRT) is widely used to treat liver tumors inducing atrophy of the treated lobe and contralateral hypertrophy. The lack of animal model has precluded further investigations to improve this treatment. We developed an animal model of liver damage and atrophy–hypertrophy complex after SIRT. Three groups of 5–8 rabbits received transportal SIRT with Yttrium 90 resin microspheres of the cranial lobes with different activities (0.3, 0.6 and 1.2 GBq), corresponding to predicted absorbed radiation dose of 200, 400 and 800 Gy, respectively. Another group received non-loaded microspheres (sham group). Cranial and caudal lobes volumes were assessed using CT volumetry before, 15 and 30 days after SIRT. Liver biochemistry, histopathology and gene expression were evaluated. Four untreated rabbits were used as controls for gene expression studies. All animals receiving 1.2 GBq were euthanized due to clinical deterioration. Cranial SIRT with 0.6 GBq induced caudal lobe hypertrophy after 15 days (median increase 34% -ns-) but produced significant toxicity. Cranial SIRT with 0.3 GBq induced caudal lobe hypertrophy after 30 days (median increase 82%, *p* = 0.04). No volumetric changes were detected in sham group. Transient increase in serum transaminases was detected in all treated groups returning to normal values at 15 days. There was dose-dependent liver dysfunction with bilirubin elevation and albumin decrease. Histologically, 1.2 GBq group developed permanent severe liver damage with massive necrosis, 0.6 and 0.3 GBq groups developed moderate damage with inflammation and portal fibrosis at 15 days, partially recovering at 30 days. There was no difference in the expression of hepatocyte function and differentiation genes between 0.3 GBq and control groups. Cranial SIRT with 0.3 GBq of ^90^Y resin microspheres in rabbits is a reliable animal model to analyse the atrophy–hypertrophy complex and liver damage without toxicity.

## Introduction

Selective internal radiation therapy (SIRT; also known as radioembolization) is an established therapy for patients with different malignancies with liver predominant disease that consists in the intra-arterial administration of radioactive microspheres. The aim of SIRT is to deliver a high dose of radiation selectively to liver tumors and spare the surrounding liver parenchyma as much as possible from the effects of radiation^1,2^. Glass or resin microspheres carrying yttrium-90 (^90^Y) have been used most extensively for this purpose^3^. The efficacy of SIRT depends on the amount of radiation absorbed by the tumor, which in turn depends on the proportion of the arterial blood supply that reaches liver tumors compared to the surrounding liver parenchyma^4^. The other main limiting factor for an effective SIRT is the relatively low tolerance of the liver parenchyma to radiation. The effects of radiation can induce changes in liver morphology, volume and function; clinical syndromes including radioembolization-induced liver disease (REILD)^5^; or portal hypertension^6^.

Lobar SIRT may induce atrophy of the treated lobe and hypertrophy of the contralateral lobe, known as atrophy–hypertrophy complex. SIRT-induced contralateral liver hypertrophy was first described in patients with colorectal liver metastasis, and confirmed in patients with HCC^6,7^. The degree of hypertrophy ranges from 29% at 6 weeks to 57% at 12 months post-treatment^8^. Contralateral hypertrophy suggests a compensatory mechanism to ipsilateral atrophy^9^, although the mechanisms underlying this atrophy–hypertrophy complex are not fully understood. Nevertheless, contralateral hypertrophy may solve the problem of an insufficient future liver remnant (FLR), which is a major limitation to extended hepatectomy^10^.

The lack of animal models has hampered the progression of SIRT for the treatment of liver cancer compared to other treatment modalities, and the development of better SIRT procedures. In this study, we describe an animal model of SIRT in rabbits that may help overcome these limitations.

## Results

### Optimization of the experimental model

**Figure 1:**
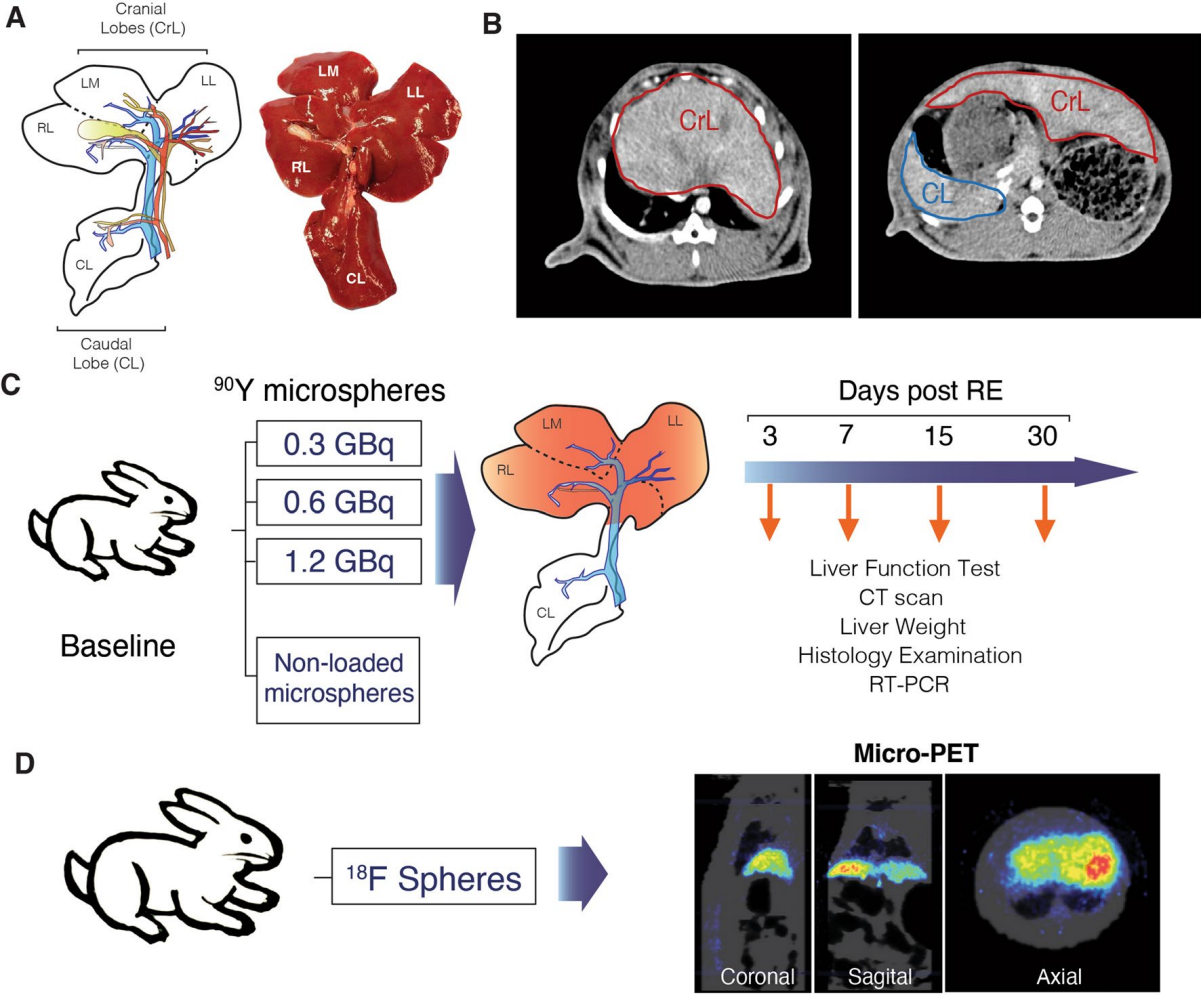
Experimental design. (**A**) Liver anatomy of the rabbit, subdivided into four main lobes. These are the caudal lobe (CL) and three cranial lobes (CrLs): the right (RL), medial left (LM) and lateral (LL) left lobes, each supplied by branches of the arterial and portal venous system. This figure is reprinted from J. Surg. Res. 2011;171:486–94^19^, with permission from Elsevier. (**B**) Axial view of a contrast enhanced CT performed in a rabbit. Manual delineation of CrLs and CL for volumetry quantification. (**C**) Experimental design. Four experimental groups of animals were treated with ^90^Y-microspheres or non-loaded microspheres. Radiation toxicity, liver function, liver volume by CT scan, histological examination and gene expression involved in inflammation, regeneration and differentiation, were evaluated at different time points, as indicated in the text. (**D**) Four animals received fluorine-18 labelled resin microspheres, to evaluate the anatomical distribution and confirm selectivity of the delivery in CrLs while sparing of the CL. MicroPET images showed deposition in the CrLs, no CL deposition neither lung activity was observed.

**Figure 2:**
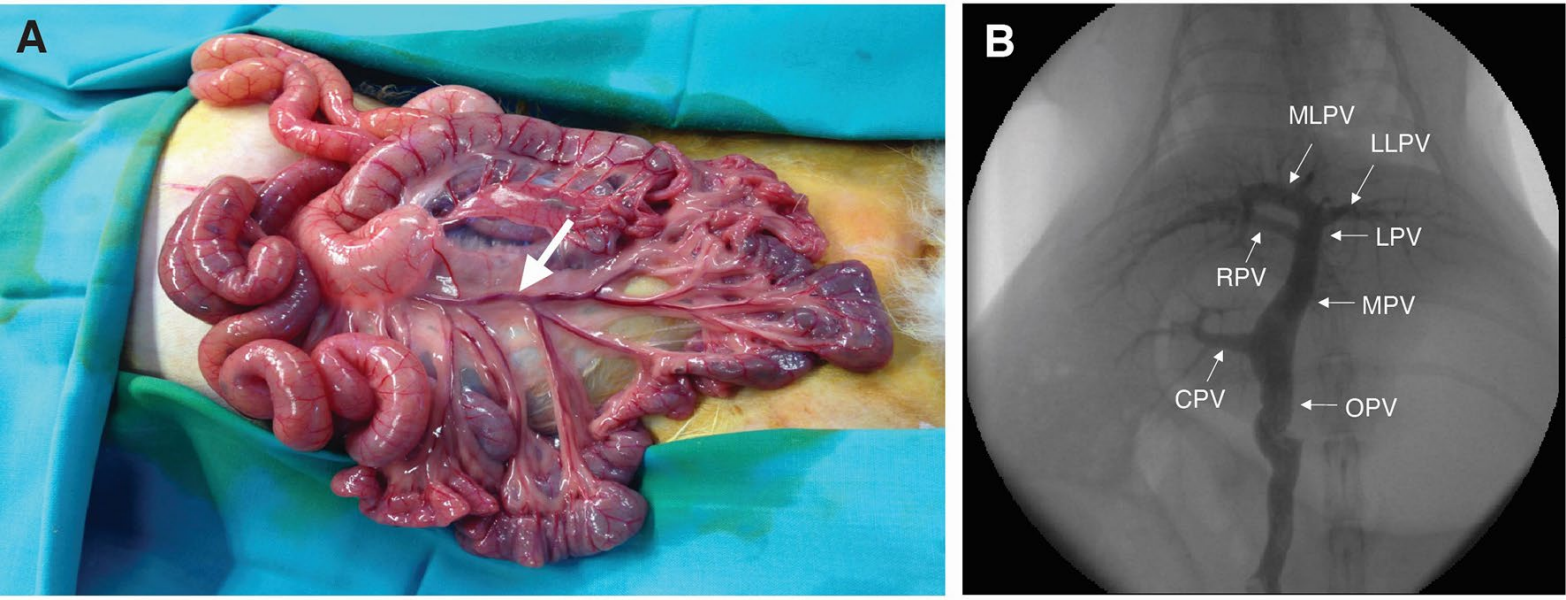
Direct portography technique and rabbit liver portal anatomy. (**A**) Exposition of the small bowel after laparotomy, showing a branch of the superior mesenteric vein (white arrow). (**B**) Direct portography shows the conventional liver rabbit portal anatomy: the original portal vein (OPV) divides into the main portal vein (MPV) and caudate portal vein (CPV). The MPV then bifurcates into the right portal vein (RPV) and left portal vein (LPV) which subsequently divided into medial left portal vein (MLPV) and lateral left portal vein (LLPV).

The animal model allowed a selective delivery of the radioactive microspheres in vivo (see Figure 1 and 2 and Materials and Methods section). Histological examination of the radiated lobes showed that microspheres were mainly located in the interstitium of portal spaces, as previously described^11^, with a heterogeneous distribution within each lobe. The density of microspheres was variable in the different CrLs. Particles were not found in the CL in any animal.

The estimates of ^90^Y activity needed to deliver target doses of radiation of 200, 400 and 800 Gy proved to be accurate enough. Using the actual CrLsV of each animal, median (IQR) predicted doses of radiation were 200 Gy (143–259 Gy) in animals receiving 0.3 GBq, 490 Gy (468–576 Gy) in those receiving 0.6 GBq, and 876 Gy (751–1126 Gy) in those receiving 1.2 GBq (Supplementary Table 1).

**Figure 3:**
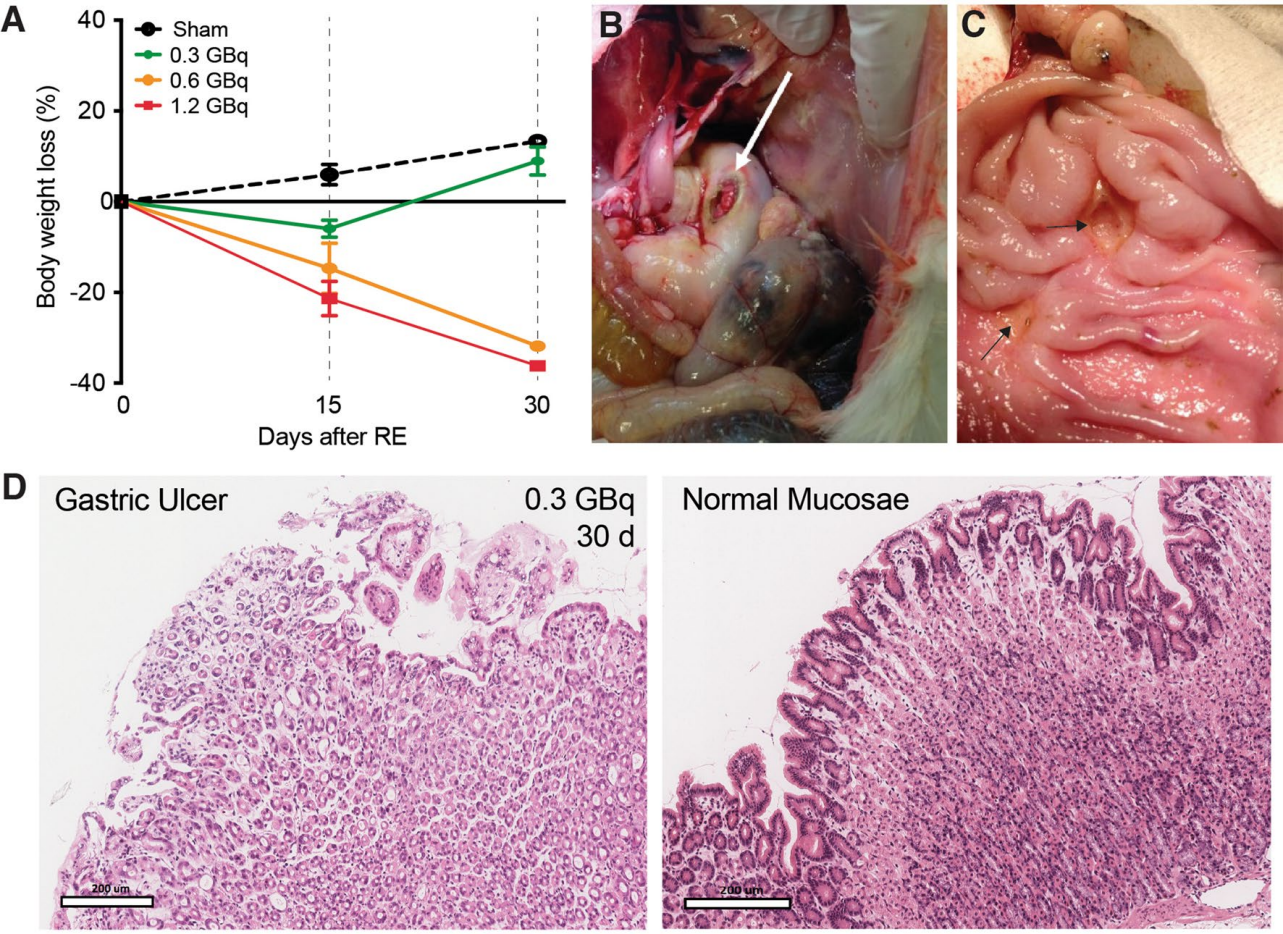
Extrahepatic toxicity of CrLs SIRT. (**A**) Body weight in different groups along the time. (**B**) Gastric mucosal ulceration with perforation (white arrow) 30 days after 0.6 GBq injection. (**C**) Superficial gastric ulcers (black arrows) in the vicinity of the irradiated CrLs 30 days after 0.3 GBq injection. (**D**) Hematoxylin & Eosin-stained sample a normal gastric mucosae and a subverted mucosae from a gastric ulcer 15 days after SIRT.

The highest ^90^Y activity resulted in lethal toxicity. All 5 animals receiving 1.2 GBq died or were sacrificed due to clinical deterioration. Fifteen days (± 3 days) after SIRT, mean weight loss from baseline in this group was 20% (Fig. 3A). Weight loss could be at least partially explained by gastric lesions found at necropsy, consisting in a firm adhesion of the stomach to the left lateral cranial lobe and macroscopic gastric ulceration.

The ^90^Y activity of 0.6 GBq also produced significant toxicity in some animals. Two out of 5 rabbits in this group died or had to be euthanized due to clinical deterioration. Mean weight loss from baseline was 15% (Fig. 3A). Three rabbits sacrificed at day 15 had mild gastric inflammation, and 2 rabbits sacrificed at day 30 had gastric ulceration, one of them with adjacent gastric perforation (Fig. 3B).

Finally, the lowest ^90^Y activity of 0.3 GBq was well tolerated. Animals in this group experienced minimal body weight loss (mean 6%) (Fig. 3A). None of the 3 animals sacrificed at day 15 had gastric lesions, and 3 out of 4 animals showed only mild inflammation and/or ulceration at day 30 (Fig. 3C, D). The animal sacrificed at 60 days did not show gastric damage.

### SIRT induced atrophy–hypertrophy complex

**Figure 4:**
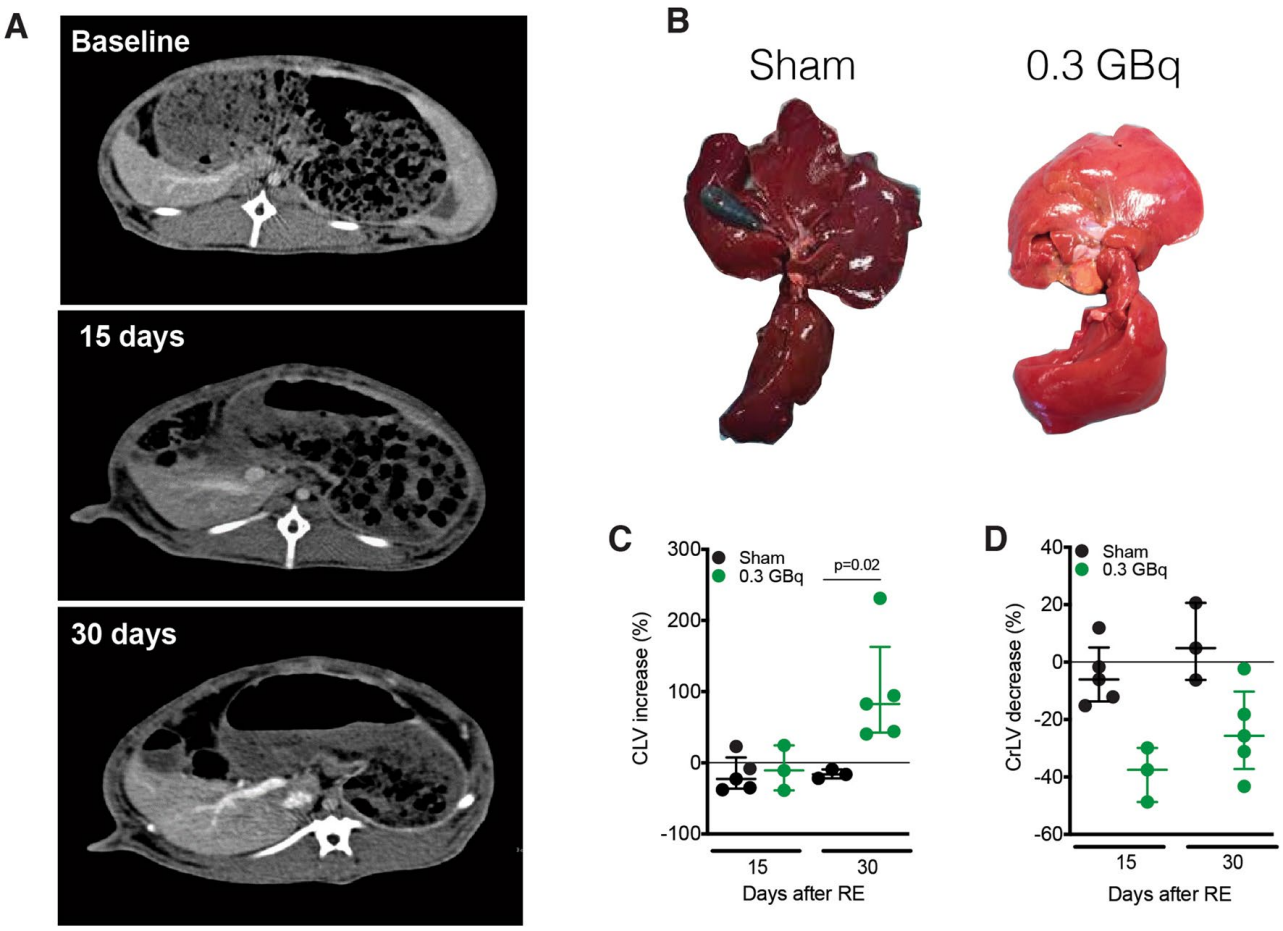
SIRT induced atrophy/hypertrophy complex. (**A**) Axial view of a contrast enhanced CT at baseline, 15 and 30 days after CrLs SIRT with 0.3 GBq, showing CL volume increase. (**B**) Macroscopic aspect of CrLs atrophy and CL hypertrophy at 30 days in 0.3 GBq group as compared with sham group. (**C**) Increase in CL volume at 15 and 30 days in Sham and 0.3 GBq group. (**D**) Decrease in CrLs volume at 15 and 30 days in Sham and 0.3 GBq group.

CrLs SIRT with 0.3 GBq induced a significant contralateral hypertrophy, with an increase in CLV after 30 days (median 82%, *p* = 0.04). Atrophy of the treated CrLs preceded the compensatory hypertrophy of CLV (Fig. 4A, B). A significant decrease in CrLsV was already present after 15 days (median 37%, *p* = 0.02) and did not progress thereafter (median decrease at day 30 of 25%). CrLs SIRT with a higher dose of 0.6 GBq induced more intense volumetric changes after 15 days. The median increase in CLV was 34% (ns) and the median decrease in irradiated CrLs was 61% (ns).

No correlation was observed in either group between the magnitude of the CLV increase and the magnitude CrLsV reduction. Importantly, the administration of cold, non-loaded microspheres in the sham group did not induce any volumetric changes after 15 or 30 days (Fig. 4C, D). Liver volumetric data from all groups at baseline and during follow-up are presented in Supplementary Table 2.

### SIRT induced liver damage

**Figure 5:**
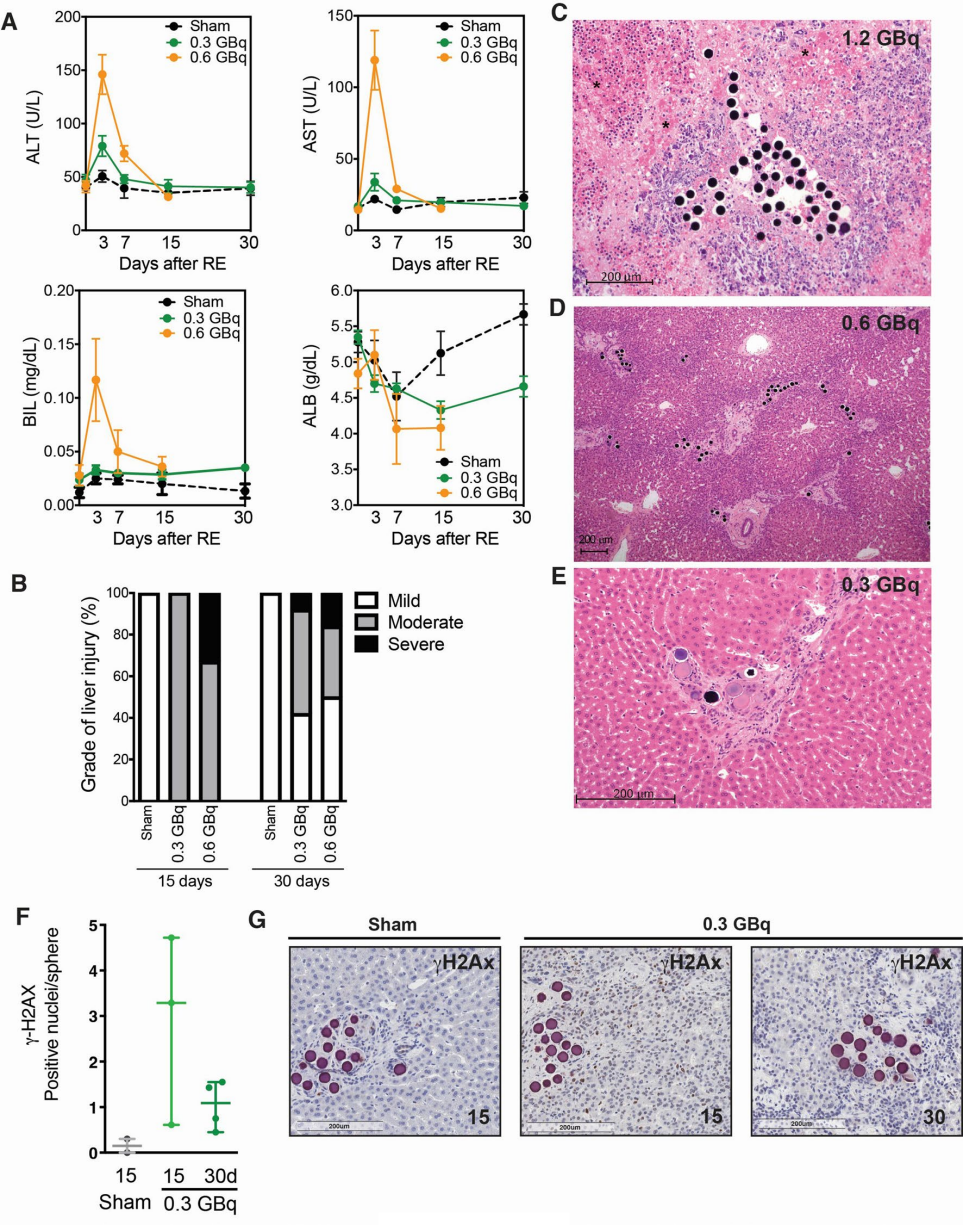
SIRT induced liver damage. (**A**) Serum liver transaminases and liver function in rabbits after CrLs SIRT. (**B**) Severity of liver damage in CrLs distribution among groups and along the time. (**C**) Severe liver damage: massive tissue necrosis (*) and periportal hepatocyte atrophy. Dense deposit of extravascular microspheres in the portal tract. (H&E, × 200). (**D**) Moderate liver damage: periportal necrosis, portal fibrosis with porto-portal fibrotic bridges, lobulillar atrophy, light ductular proliferation and dense extravascular particle concentration in portal tracts (H&E × 100). (**E**) Mild liver damage: foreign body giant cell reaction, frequent binucleate hepatocytes and slight portal lymphocytic infiltrate with preserved liver architecture. (H&E, × 200). (**F**) Quantification of DNA damage expressed as number of Gamma-H2AX-positive nuclei and (**G**) representative Gamma-H2AX stainings in CrLs in 0.3 GBq in comparison with sham group at 15 and 30 days.

CrLs SIRT induced a dose-dependent and transient increase in transaminases in all groups, returning to normal values on day 15 (Fig. 5A). These differences were not statistically significant, probably due to the small number of animals in each group. Similarly, there was a dose-dependent liver dysfunction with bilirubin elevation and albumin decrease. These alterations were unremarkable in 0.3 GBq group (actual absorbed dose of 200 Gy). Transaminases and liver function remained stable in the sham group.

Different grades of histological liver injury were observed after CrLs SIRT (Supplementary Table 3), and the severity was related to the absorbed dose of radiation (Fig. 5B). Administration of 1.2 GBq (actual absorbed dose of 876 Gy) induced severe liver damage in all animals at days 15 and 30, with massive necrosis (Fig. 5C). Administration of 0.6 GBq (actual absorbed dose of 490 Gy) and 0.3 GBq (200 Gy) induced moderate liver damage after 15 days in most animals, with foreign body giant cell reaction, inflammation and periportal necrosis, portal fibrosis and porto-portal bridges (Fig. 5D). After 30 days, nearly half the CrLs receiving 0.3 or 0.6 GBq showed only mild liver damage (Fig. 5E). This suggests that liver damage occurs early after SIRT and recovers at later stages in parallel with the time course of biochemical alterations. The sham group showed mild histological alterations as slight portal lymphocytic infiltrate and giant cell reaction at days 15 and 30. According to this finding, DNA damage was confirmed by gamma-H2AX immunostaining in CrLs with 0.3 GBq 15 days after SIRT, with a trend to recover at 30 days (Fig. 5F, G).

**Figure 6:**
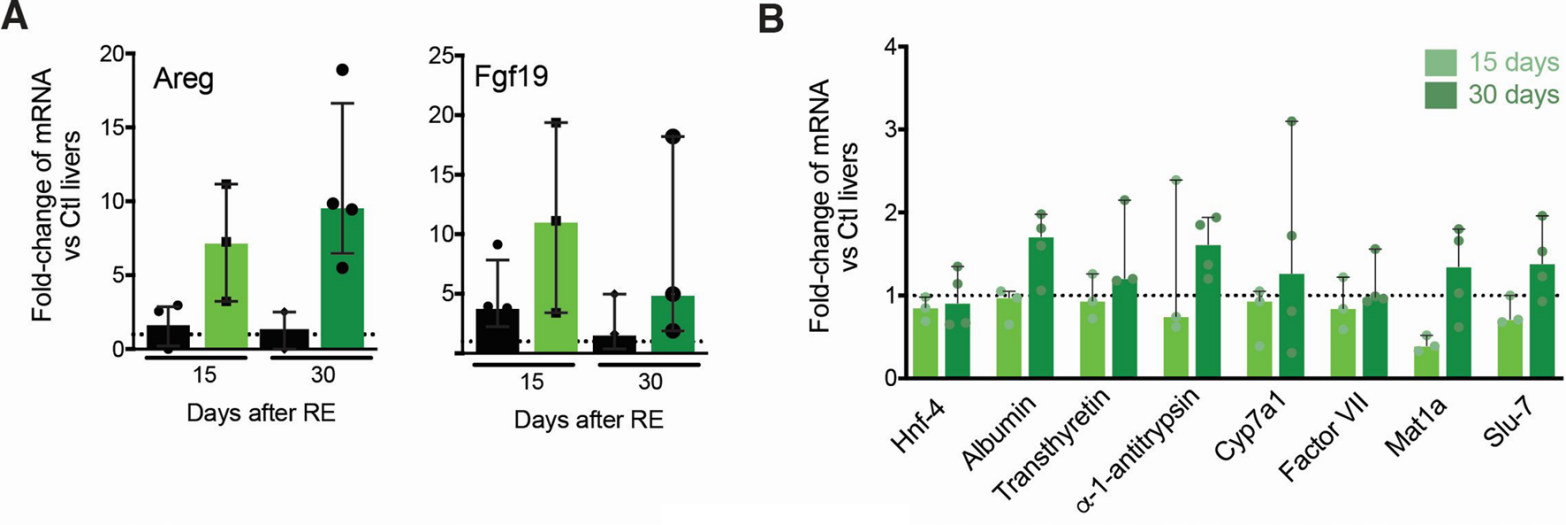
Gene expression. (**A**) Gene expression of regenerative cytokines in the CrLs in sham (black bar) and 0.3 GBq group (green bar) as compared with controls (dot line). (**B**) Gene expression of hepatocyte-specific proteins in the CrLs in 0.3 GBq group (green bar) as compared with controls (dot line).

Gene expression of inflammatory cytokines involved in early liver damage (IL-6, IL-1b) showed a mild increase in sham and 0.3 GBq groups at days 15 and 30 (Supplementary Figure 1) and a higher increase in Areg and FGF-19, suggesting a regenerative stimulus (Fig. 6A). To assess the impact on hepatocyte function and differentiation, we analysed the expression of hepatocyte-specific genes including albumin, HNF-4, MAT1a, splicing factor SLU7, transtirretine, alpha1 antitrypsine, Cyp7a1, factor VII. Thirty days after CrLs SIRT with 0.3 GBq, no difference in the expression of any of these was observed between treated and control animals, suggesting that hepatocyte function is maintained after CrLs SIRT at this activity (Fig. 6B).

**Figure 7:**
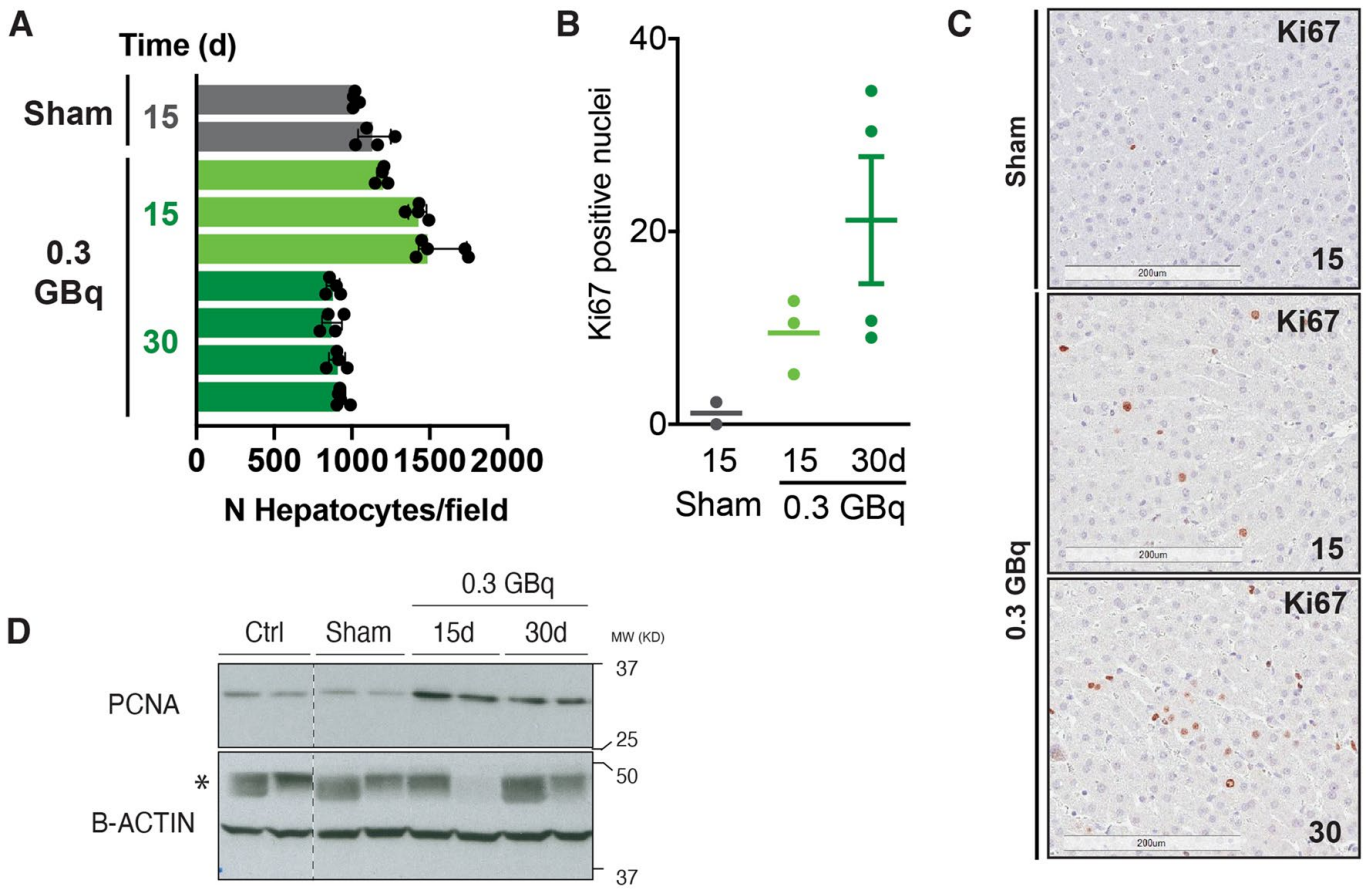
Liver hyperplasia in CL after CrLs SIRT with 0.3 GBq (200 Gy) at 15 and 30 days. (**A**) Quantitative hyperplasia in 0.3 GBq in comparison with sham group. (**B**) Quantification of Ki67-positive nuclei and (**C**) representative Ki67 stainings in CL in 0.3 GBq and sham group. (**D**) PCNA and B-Actin protein expression in 0.3 GBq, sham and control group. *MW* molecular weight, *KD* kilodaltons, *unspecific band.

CrLs SIRT with 0.3 GBq induced liver hyperplasia in the CL at day 15 (Fig. 7A). In agreement with this finding, enhanced hepatocellular proliferation as assessed by Ki-67 staining (Fig. 7B, C) and higher levels of PCNA were found in the CL 15 and 30 days after RE (Fig. 7d) (Full length blots are presented in Supplementary Figure 2).

## Discussion

SIRT is widely used to treat primary liver tumours and liver metastasis from colorectal, neuroendocrine and other tumors^12^. While the ability of SIRT to induce objective tumor responses is well-known, its capacity to produce clinical and subclinical liver damage is less recognized. However, it limits the ability to deliver high doses of radiation when the whole liver is targeted or when the liver bears pre-existing damage due to cirrhosis or prior therapies^13^. Indeed, SIRT frequently results in some degree of liver atrophy^9^ that may or may not have clinical consequences. In lobar SIRT, atrophy of the treated lobe is commonly followed by hypertrophy of the contralateral lobe. When this phenomenon of atrophy–hypertrophy complex occurs in the setting of tumour growth control or response, the result is a chance to rescue for surgery patients that were unresectable on the basis of a low FLR. Comprehension of the pathogenic mechanisms leading to atrophy and hypertrophy may help develop treatments or procedures to hamper the former or enhance the latter, to make SIRT a better therapeutic tool.

Animal disease models are important tools to better understand human disease processes and to design and refine medical therapies. Opposite to drugs, medical devices often require models involving large animals and complex procedures. We have developed a consistent animal model of atrophy–hypertrophy complex after CrLs SIRT that may help address some of the relevant issues of SIRT summarized above. Until very recently there was not any animal model for lobar SIRT. A recently reported rat model^14^ replicates SIRT induced atrophy but not contralateral hypertrophy. The rabbit model is in general more suitable for locoregional therapies based on their more favourable liver anatomy and the possibility to use the same catheters as for humans. It has been widely used as a model for portal vein embolization^15^. For our purpose, having the CL separated from the three CrLs allows to measure liver volumes separately and accurately^16^.

An interesting aspect of our model is the administration of radioactive microspheres through the portal vein. In the absence of a highly vascularized liver tumour like in the Vx2 model, the rabbit hepatic arteries are just too thin to use a microcatheter without inducing severe spasm. Vascular spasm precludes the injection of the number of microspheres that is needed to administer even a small amount of resin microspheres. Contrary, transportal administration of ^90^Y microspheres was technically easier for an experienced interventional radiologist. It had been performed almost 3 decades ago^17^, but microspheres were administered into the main portal vein of the rabbits by injection through a jejunal vein. For an animal tumour model intended to improve the antitumor efficacy of SIRT, the intraportal route would have not be an option, although it has been described in a single patient with HCC^18^. For a model to study liver injury, the end result is comparable as shown by the histological characterization of the liver lesions in our model. Hence, this study provides a solid and reproducible model of lobar SIRT, which allows investigating the pathophysiological mechanisms involved in SIRT-induced liver damage, and potential drugs or strategies to reduce liver damage or to enhance contralateral hypertrophy.

One of the strengths of our model is the robustness of the SIRT procedure. All animals were treated by the same experienced radiologist. In all animals, it was verified before sacrifice that the cranial portal vein was permeable, and in 4 animals the selectivity of the distribution of the spheres in the cranial segments, with no activity in the caudal lobe, was verified in a small-animal imaging PET scanner. Another quality control of the robustness of the model is the excellent correlation between CT volumetry at time of sacrifice and liver weight measured after sacrifice (Spearman correlation coefficient, r = 0.89). This similar concordance has been also described by other authors in a rabbit model for selective portal embolization^19^. Finally, the intraobserver correlation in two measurements of the liver lobes volumes by CT is also excellent.

Because of the scattered deposition of the sources of radiation in SIRT compared to the uniform doses delivered by external beam irradiation, the liver tolerates higher doses of radiation in the former. In humans, the general recommendation is not to deliver an average dose of radiation in excess of 40–60 Gy to the non-tumoral liver tissue^20^. The rabbit liver is more resistant to radiation than the human liver. We have observed a relationship of both atrophy and hypertrophy with the dose of radiation, a dose–response effect recently demonstrated also in patients^21^. Doses in the range of 200 Gy produced marked liver atrophy that was nevertheless tolerated and later compensated by contralateral hypertrophy. In this regard, our model reproduces the atrophy–hypertrophy complex described in humans, where clinically tolerable irradiation results in a relevant atrophy–hypertrophy complex. This contralateral lobe hypertrophy could be secondary to both hepatocyte hyperplasia and hypertrophy as the degree of hepatocyte hyperplasia was similar at days 15 and 30, thus suggesting that hepatocyte hypertrophy also contributes to progressive contralateral lobe enlargement.

It is worth noting that marked atrophy in our model occurred with only mild and transient elevation of transaminases, imitating here too what happens in patients^22^. And liver function was also preserved despite atrophy. In fact, the expression of liver-specific genes at day 15 and 30 was already similar to controls in the 0.3 GBq group, suggesting a rapid functional recovery of the irradiated liver.

We hypothesize that a persistent subacute reduction in blood flow to the treated lobe may be behind this “silent” atrophy. This impact on liver blood flow, if it exists, should be caused by tissue changes induced by radiation and not by microembolization since animals that received cold spheres did not experience significant volume changes. We have not observed endothelial damage or features of sinusoidal obstruction syndrome in our model as described in humans^5^, not even in the 0.6 GBq group. To further dissect this hypothesis and search for early liver responses and changes was beyond the scope of this study.

Animals receiving a high dose of radiation developed marked weight loss and severe gastric toxicity. Gastric lesions were likely a bystander effect caused by the radiation reaching the stomach from the neighbouring liver lobes, since microspheres were not found in gastric samples. In humans, gastric lesions are secondary to the unintended delivery of microspheres to the gastrointestinal tract through unnoticed collateral vessels^23^. In a previous study aimed to examine the radiation effect to the gastric wall from left hepatic lobe SIRT, none of 97 patients treated exhibited gastrointestinal ulcer or other grade 3–4 radiation-related GI toxicity (pain, nausea or vomiting)^24^. Yet, the average dose absorbed by the left lobe was 109 Gy in this series and the possibility of having bystander irradiation of adjacent organs after radiation segmentectomy, where the average dose of radiation can be as high as 500 Gy, cannot be ruled out. A higher sensitivity of the rabbit gastric mucosa may not be discarded either since animals in the 200 Gy group also showed subclinical gastric damage 1 month after SIRT.

Our study has a number of limitations. One is the relatively low number of rabbits studied, a result of the expensive and time-consuming management of rabbits compared to smaller rodents. The late times at which the animals were sacrificed have not allowed us to identify the onset of liver regeneration mechanisms, which may occur very early after damage, even in humans^9^. Another limitation is the absence of post-treatment real dosimetry based on PET-^90^Y voxel-based, which is of major interest in clinical SIRT induced atrophy–hypertrophy complex^25^.

In summary, CrLs SIRT with ^90^Y resin microspheres in rabbits is a reliable animal model to analyse liver injury and the atrophy–hypertrophy complex that allows translational studies to further improve the outcome of liver cancer patients treated with SIRT.

## Materials and methods

### Animals

Adult female New Zealand white rabbits (San Bernardo Farm, Spain) with a mean weight of 3069 g ± 188.5 g were used. They were acclimatized for at least 7 days under standardized laboratory conditions in a temperature-controlled room with a 12 h light/dark cycle and with access to standard chow and water ad libitum.

The rabbit was chosen since it is considered the laboratory animal best suited for evaluating liver-directed therapies. The rabbit liver is subdivided into four main lobes: the caudal lobe (CL) and three cranial lobes (CrLs): right, medial left and lateral left, each supplied by branches of the arterial and portal venous systems^16^ (Fig. 1A). As the CrLs are isolated from the CL, the rabbit is ideally suited for selective intravascular therapies. Indeed, the CL and the CrLs are separated by the stomach, thus allowing to study lobar volumes independently by computed tomography (CT)^16^ (Fig. 1B).

All applicable institutional and national guidelines for the care and use of animals were followed. The experimental protocol was approved by the Institutional Animal Care and Use Committee of the University of Navarra. This study followed the ARRIVE guidelines.

### Experimental design

There were 4 experimental groups of animals. Three groups of 5–8 animals received SIRT to the CrLs (CrLs SIRT) with three different target doses of absorbed radiation (200, 400 and 800 Gy) through the administration of ^90^Y-resin microspheres (SIR-Spheres; Sirtex Medical Ltd, Sydney, Australia) and one group received cold, non-loaded microspheres (sham group). ^90^Y-loaded and cold microspheres were generously donated by Sirtex Medical. Changes in lobar volumes, liver biochemistry, histopathology, and molecular expression of genes involved in inflammation, regeneration and differentiation, were evaluated at different time points (Fig. 1C). Four untreated rabbits were used as controls for gene expression studies.

#### Activity calculation

Yttrium-90 activity to be administered was calculated according to the Partition Model equation^26^, assuming a uniform deposition of ^90^Y microspheres in the CrLs tissue and absence of lung shunting. In brief, the activity that results in a target dose of absorbed radiation is proportional to the targeted liver volume. As an average, the CrLs of a 3000 g New Zealand rabbit weight 67 g, so the activity needed to deliver the intended doses of 200, 400 and 800 Gy were 0.3, 0.6 and 1.2 GBq of ^90^Y resin microspheres, respectively.

To analyse the effects of microspheres in the absence of radiation, the sham group received non-^90^Y-loaded microspheres in amounts equal to those used to administer 0.3 GBq (n = 3) and 1.2 GBq (n = 2).

#### Anatomic distribution

To assess anatomical distribution, selective delivery to CrLs, and to avoid safety concerns related to the manipulation of ^90^Y, fluorine-18-labeled resin microspheres were studied in 4 rabbits^13^ with a microPET. Briefly, after the decay of ^90^Y, the resin microspheres were pelleted by centrifugation and incubated with fluorine-18 for 15 min at room temperature with gentle mixing. The resin microspheres were rinsed with a mixture of PBS and saline several times until the activity in the supernatant was negligible. Finally, the microspheres were suspended in saline for injection. PET imaging was performed in a dedicated small animal tomography (Mosaic, Philips), with 2 mm resolution, and 11.9 cm and 12.8 cm axial and transaxial field of view respectively. Images were reconstructed using the 3D Ramla algorithm (a true 3D reconstruction) with 2 iterations and a relaxation parameter of 0.024 into a 128 × 128 matrix with a 1 mm voxel size applying dead time, decay, random, scattering and attenuation corrections (Fig. 1D).

Experimental design is shown in Supplementary Table 1.

### SIRT procedure

Rabbits were monitored and supervised by specialized nurses. For anaesthesia, each rabbit was given an intramuscular injection of 10 mg/kg ketamine (Merial), 0.15 mg/kg of medetomidine (Esteve Veterinaria) and 2–8 mg/kg of intravenous propofol (Braun). The animals were placed in supine position and 1.5–3% sevoflurane (Baxter) served to maintain anaesthesia. A laryngeal mask size 1 was used and 0.5 mg/kg of atracurium besilate (GlaxoSmithKline) was used as muscle relaxant. During the procedure, animals received an intravenous perfusion of 10–20 mcg/kg/h of either fentanyl (Kern Pharma) or 0.6 mcgr/kg/min of remifentanyl (GlaxoSmithKline). Before and 5 days after the procedure, 0.01–0.05 mg/kg of Buprenorfin (Grunenthal Pharma) and 5 mg/kg of enrofloxacin (Karizoo) were administered subcutaneously. Omeprazol (Normon) was administered at 2 mg/kg intravenously during the procedure, and 20 mg/day orally for 5 days after procedure.

A transarterial administration of radioactive microspheres was initially attempted. The common right femoral artery was exposed and catheterized with a 4F introducer. The hepatic arteries were then catheterized with different microcatheters once a 4F Cobra catheter (Cordis Corporation) was placed in the celiac trunk. However, the size of the hepatic arteries was not large enough to inject all the microspheres needed to deliver the intended amount of radioactivity in 5 consecutive animals. This technical issue, and the fact that is a non-tumoral animal model, led to the design of a procedure for transportal delivery.

A midline laparotomy was made from the epigastrium down for a length of 8–10 cm. After exposure of a small bowel loop, a small branch of the superior mesenteric vein (SMV) was punctured with a 24-gauge needle (Abbott Laboratories) (Fig. 2A). The stylet of the needle was removed and a 0.014´´ guidewire (Boston Scientific MediTech) was advanced with fluoroscopic guidance towards the liver. A 4F coaxial introducer (Cook Medical) was advanced into the SMV. Direct portography was performed by hand injection of 3–5 mL of iodinated contrast (Radialar 280 mg/ml, Juste SAQF) (Fig. 2B).

After a detailed anatomical study of the portal venous system of the rabbit, a 2.7 F microcatheter (Terumo) was advanced over its wire. The tip was set in the distal third of the main portal vein, distal to the origin of the caudal portal vein. The standard administration equipment set (SIR-Sphere^TM^, Sirtex Medical) was used to perform the SIRT procedure. The infusion started once the microcatheter was positioned at the treatment site and the integrity of the delivery system was verified. Microspheres were delivered under direct angiographic control using 5% glucose to pulse push (30–150 ml according to the amount of spheres) and small aliquots of iodinated contrast. Correct microcatheter positioning and absence of reflux were confirmed during the procedure by fluoroscopy and repeated contrast injections. Hand-injection of ^90^Y-microspheres through the microcatheter was slow, duration ranging between 10 and 30 min depending on the number of microspheres. After the complete delivery, a portography from the 4F introducer sheath was repeated to confirm the patency of the CrLs portal vein.

When the procedure was finished and the introducer was removed, the SMV was ligated at the site of catheterization and the puncture site was covered with an absorbable hemostatic agent (Ethicon). Absence of bleeding was confirmed and the linea alba was closed with a synthetic absorbable material. The skin was closed using an interrupted pattern.

Animals receiving the highest amount of microspheres (1.2 GBq) were given 1.5 mg/kg of furosemide (Sanofi Aventis) after microspheres infusion to avoid eventual complications due to volume overload.

At the end of the study period (15 or 30 days), a new portography using the same technique above described was performed immediately before sacrifice, to confirm the patency of the cranial portal branches. Animals were euthanized by intravenous injection of 0.3 ml/kg of a cocktail containing embrutamide, iodide of mebenzonium and tetracaine hydrochloride (T61).

### Contrast-enhanced CT acquisition protocol and CT volumetry

All examinations were performed with a 64 multidetector computed tomography (Somatom Sensation, Siemens Medical Systems). Examination parameters were 64 x 0.6 mm collimation, 1.4 mm/s table feed, 2 mm section thickness, 1.5 mm reconstruction interval, 80 kV, 65 mA, and 0.5 s rotation time. Rabbits were sedated by intramuscular injection of 10 mg/kg ketamine and 0.15 mg/kg of medetomidine and were placed in supine position. After unenhanced scan acquisition, a contrast enhanced scan was performed 15 s (arterial phase), 30 s (portal phase), 45 s (venous phase), and 60 s (late venous phase) after intravenous injection in the marginal ear vein of 4 mL non-ionic contrast agent (Iohexol, 300 mg/ml; Omnipaque, GE Healthcare), followed by 3 mL of saline solution. Three-dimensional reconstructions of the liver were composed by superimposing sequential reconstructed 2-mm axial images in an external workstation (Leonardo, Siemens Healthcare).

Total liver volume (TLV), caudal lobe volume (CLV) and cranial lobes volume (CrLsV) were calculated for each animal. The outline of the region of interest was traced manually in each image section. Volumetric values were obtained multiplying the sum of delineated areas by the section thickness. Increase in CLV was expressed as percent of the basal baseline value and was calculated using the formula:

$${\text{Increase CLV:}}\;\left[ {({\text{CLV post-RE}} - {\text{CLV pre-RE}}){\text{/CLVpre-RE}}} \right] \times 100$$

A similar formula was used to obtain the decrease in CrLsV.

$${\text{Decrease CrLsV:}}\;\left[ {({\text{CrLsV post-RE}} - {\text{CrLsV pre-RE}}){\text{/CrLsVpre-RE}}} \right] \times 100$$

Two methods were used to validate volumetric measurements. First, CLV was correlated with CL weight at the time of sacrifice. Second, CLV was measured twice by the same radiologist in a blind fashion in 34 animals to evaluate intraobserver agreement. CLV measured at sacrifice correlated well with CL weight measured immediately after sacrifice (Spearman’s r = 0.89, *p* = 0.01). Repeated measurements by the same radiologist also showed an excellent correlation (Spearman’s r =  0.99, *p* < 0.001) (Supplementary Figure 3 a-b). The predicted absorbed dose of radiation was recalculated using the actual CrLsV for each animal.

### Liver damage and safety profile

Serum AST and ALT, albumin and bilirubin serum levels were determined in blood samples collected from the marginal ear artery at baseline and 3, 7, 15 and 30 days after SIRT using a chemistry analyzer (Cobas c311, Roche). Animal body weight was recorded weekly until sacrifice to detect clinical deterioration. Animals were euthanized when body weight loss reached 20% of baseline.

### Histological examination

Liver tissue samples of the CL and the three independent CrLs (right, medial left and lateral left lobes) were obtained immediately after sacrifice in all groups studied. Samples were fixed in buffered formalin, dehydrated in 70% ethanol, embedded in paraffin, and cut into 3-μm sections. Additional liver samples were snap frozen in liquid nitrogen and stored at −80 °C.

Liver specimens stained with hematoxylin and eosin (H&E) were examined by an experienced pathologist. In addition to recording the presence and localization of microspheres, the following histological features were quantified in each of the three CrLs: foreign body giant cell reaction, inflammation (portal/lobular), necrosis, fibrosis (periportal fibrosis/portoportal bridges), lobular atrophy and ductular proliferation. According to the presence and the intensity of these changes, CrLs damage was graded as mild, moderate or severe (Supplementary Table 3).

To quantify the degree of liver hyperplasia in CL, the number of hepatocytes per unit area of ​​the CL were counted in H&E stained slides by customizing a Fiji script (imageJ, NIH). The pathologist selected manually a region of interest (ROI) of 2500 × 2500 pixels. An algorithm was designed to recognize the nuclei of hepatocytes according to their size and shape, and was then manually supervised to discard nuclei of Kupffer cells, bile duct cells, sinusoidal cells and endothelial cells. A total of 4 fields of a histological section stained with H&E were studied, which means a total area analysed per case of 1.58 mm^2^.

Cell proliferation in the CL was estimated by mean of Ki67 and PCNA. Ki67 immunostaining in paraffin tissue was performed using mouse monoclonal antibody from Novocastra (NCL-L-Ki67-MM1)^27^. For quantification of Ki67-positive cells, images were captured at 20 × magnification (Aperio CS2, Leica) and 10 arbitrarily chosen fields were counted using ImageJ software (NIH, Bethesda, Maryland, USA). For estimating DNA damage in the CrL, gamma H2A.X immunohistochemical staining was performed using the EnVision^TM^+ System (Dako, K400111-2; Glostrup, Denmark) according to the manufacturer’s recommendations. Paraffin sections were cut, dewaxed and hydrated. Antigen retrieval was performed for 30 min at 95 °C in 0.01 M Tris-1 mM EDTA buffer (pH 9) in a Pascal pressure chamber (S2800, Dako). Endogenous peroxidase was blocked with 3% H_2_O_2_ and sections were incubated with anti-gamma H2A.X (1:1000, Novus Biologicals, NB100-74435). Then, sections were incubated with goat anti-mouse labelled polymer EnVision^TM^+ System (Dako, K400111-2; Glostrup, Denmark) for 30 min at room temperature and peroxidase activity was revealed using DAB+ (Dako, K346811-2). Finally, sections were lightly counterstained with Harris hematoxylin, dehydrated, and coverslipped with Eukitt (Labolan, 28500).

### Western Blot analysis

Frozen liver samples were lysed in RIPA buffer. Protein extracts were subjected to western blot analysis as reported^28^. Briefly, immunoblotting analyses were carried out in 10%SDS-PAGE, loading 30 µg of protein per lane and transferred onto nitrocellulose membranes (GE Healthcare, Buckinghamshire, UK). Primary antibodies against PCNA (1:1000) (Santa Cruz Biotechnology, Cat sc-56) and B-Actin (1:2000) (Sigma Aldrich, Cat A2066) were diluted in TBS-tween with 5% milk. Anti-mouse (Pierce, Cat 31430) and anti-rabbit (Cell Signaling, Cat 7074) IgG HRP-linked secondary antibodies were diluted 1:5000. Bands of immunoreactive proteins were visualized using enhanced chemiluminescence (ECL, Perkin Elmer, Waltham, USA).

### RNA isolation and quantitative-PCR

Gene expression of cytokines and growth factors was analysed in CrLs and CL samples obtained immediately after euthanasia. Total RNA from liver tissue was extracted using the automated Maxwell system from Promega (Madison, WI, USA), and then reverse transcribed to cDNA as previously described^28^. Real-time PCRs were performed with iQ SYBR Green supermix (Bio-Rad, Hercules, CA) in an iQ5 thermocycler from Bio-Rad as described^28^. Quantification was represented as expression units, i.e. 2^CT * 1000, being CT the difference in number of cycles between GAPDH expression and that of the measured gene. Oligonucleotides for RT PCR are described in Supplementary Table 4.

### Statistical analysis

Data are presented as mean and standard error of the mean (SEM). Dependent related variables were compared using Wilcoxon test and unrelated samples were compared using Mann–Whitney U test. Correlations were studied using the Spearman test. The significance was established at *p* < 0.05. Statistical analysis was performed with SPSS 20 (SPSS Inc., Chicago, IL).

## Supplementary Information

Supplementary Information.
