# Supplementary material for: A new animal model of atrophy–hypertrophy complex and liver damage following Yttrium-90 lobar selective internal radiation therapy in rabbits
Source: Sci Rep. 2022 Feb 2;12:1777. doi: 10.1038/s41598-022-05672-3 (PMC8810801; doi:10.1038/s41598-022-05672-3)

**Supplementary Table 1. Experimental design**

| **Group** | **Intervention** | **Microspheres** | **Prescribed ^90^Y activity** | **Planned absorbed dose of radiation to the CrLs** | **Actual absorbed dose of radiation to the CrLs** |
| --- | --- | --- | --- | --- | --- |
| 0.3 GBq group | Transportal  administration | ^90^Y microspheres | 0.3 GBq | 200 Gy | 200 Gy |
| 0.6 GBq group | Transportal  administration | ^90^Y microspheres | 0.6 GBq | 400 Gy | 490 Gy |
| 1.2 GBq group | Transportal  administration | ^90^Y microspheres | 1.2 GBq | 800 Gy | 876 Gy |
| Sham group | Transportal  administration | Non-^90^Y -loaded microspheres | NA | NA | NA |
| Fluor group | Transportal  administration | 18-F-loaded microspheres | NA | NA | NA |
| Control group | No | NA | NA | NA | NA |

NA: not applicable; GBq: Gigabequerel; Gy: Grays

**Supplementary Table 2: Basal CT volumetry before and after SIRT at 15 and 30 days, in sham group, and in 0.3 and 0.6 GBq groups**

| **Group** | **Pre-RE volume (cc)** | | | **CT scan**  **(days)** | **Post-RE volume (cc)** | | | **CLV increase**  **(%)** | **CrLsV decrease**  **(%)** |
| --- | --- | --- | --- | --- | --- | --- | --- | --- | --- |
|  | **CLV** | **CrLsV** | **TLV** |  | **CLV** | **CrLsV** | **TLV** |  |  |
| Sham  n=5 | 29,1 | 96,20 | 125,30 | 15 | 18,9 | 81,6 | 100,5 | -35,05 | -15,17 |
|  | 48,5 | 97,80 | 146,30 | 15 | 30,2 | 91,9 | 122,1 | -37,73 | -6,03 |
|  | 23,9 | 105,30 | 129,20 | 15 | 18,5 | 92,5 | 111 | -22,59 | -12,15 |
|  | 15,00 | 63,00 | 78,00 | 15 | 19,00 | 71,00 | 90,00 | 26,67 | 12,70 |
|  | 22,00 | 73,00 | 95,00 | 15 | 20,00 | 71,00 | 91,00 | -9,09 | -2,74 |
|  | 29,10 | 96,20 | 125,30 | 30 | 24,40 | 90,20 | 114,60 | -16,15 | -6,23 |
|  | 48,50 | 97,80 | 146,30 | 30 | 37,90 | 117,90 | 155,80 | -21,85 | 20,55 |
|  | 23,90 | 105,30 | 129,20 | 30 | 21,70 | 110,50 | 132,20 | -9,20 | 4,94 |
| 0.3 GBq  (200 Gy)  n=8 | 26,40 | 70,60 | 97,00 | 15 | 16,20 | 49,50 | 65,70 | -38,64 | -29,89 |
|  | 23,70 | 81,90 | 105,60 | 15 | 29,50 | 42,00 | 71,50 | 24,47 | -48,72 |
|  | 21,20 | 81,90 | 103,10 | 15 | 18,90 | 51,20 | 70,10 | -10,85 | -37,48 |
|  | 19,10 | 62,50 | 81,60 | 30 | 34,90 | 43,00 | 77,90 | 82,72 | -31,20 |
|  | 27,80 | 103,10 | 130,90 | 30 | 40,10 | 58,55 | 98,65 | 44,24 | -43,21 |
|  | 18,00 | 72,00 | 90,00 | 30 | 35,00 | 58,90 | 93,90 | 94,44 | -18,19 |
|  | 11,20 | 57,10 | 68,30 | 30 | 37,10 | 55,80 | 92,90 | 231,25 | -2,28 |
|  | 16,30 | 66,10 | 82,40 | 30 | 22,90 | 49,10 | 72,00 | 40,49 | -25,72 |
| 0.6 GBq  (490 Gy)  n=3 | 15,70 | 61,80 | 77,50 | 15 | 32,10 | 14,50 | 46,60 | 104,46 | -76,54 |
|  | 28,70 | 52,60 | 81,30 | 15 | 32,40 | 28,40 | 60,80 | 12,89 | -46,01 |
|  | 23,80 | 63,00 | 86,80 | 15 | 31,90 | 24,70 | 56,60 | 34,03 | -60,79 |

**Supplementary Table 3. Graduation of liver damage according to presence and intensity of particular histological changes**

| Liver damage | Foreign body cell reaction | Portal/periportal  inflammation | Necrosis | Portal/periportal  fibrosis | Parenchymal atrophy | Ductular proliferation |
| --- | --- | --- | --- | --- | --- | --- |
| Mild | + | + | - | - | - | - |
| Moderate | + | ++ | + | + | + | + |
| Severe | ++ | + | ++ | ++ | ++ | ++ |

**Supplementary Table 4: Primers used for qPCR of rabbit mRNAs**

| **Gene** |  | **Sequence** |
| --- | --- | --- |
|  |  |  |
| ***Areg*** | Forward | 5´-TGCTGATCCTCGGCTCAGGT-3´ |
|  | Reverse | 5´-GCCAGACACTTGTGGTTCAT-3´ |
|  |  |  |
| ***Fgf19*** | Forward | 5´-TCAGCTCCGGCTACAACGT-3´ |
|  | Reverse | 5´-TGCTTGGCACTGCTCAGAGA-3´ |
|  |  |  |
| ***Hnf-4*** | Forward | 5´-GCGTGGAGGCAGGGGGAGAAT-3´ |
|  | Reverse | 5´-AAGCCCTTGCAGCCATCGCA-3´ |
|  |  |  |
| ***Albumin*** | Forward | 5´-TGGCACAATGAAGTGGGTAA-3´ |
|  | Reverse | 5´-CATAGGTGTCACGAAGACTTGG-3´ |
|  |  |  |
| ***Transthyretin*** | Forward | 5´-GCTGGACTGGTATTTGTGTCTG -3´ |
|  | Reverse | 5´-CCACCTCTGCATATTCATGG -3´ |
|  |  |  |
| ***α1antitrypsin*** | Forward | 5´-CGAGAACACCAAGGAAGAGG -3´ |
|  | Reverse | 5´-GACCTGAAGCTGCTTTTTGC -3´ |
|  |  |  |
| ***Cyp7a1*** | Forward | 5’-ATTCCATATCTGGGCTGTGC -3’ |
|  | Reverse | 5’-CCATCTCTTGGGTCAATGCT -3’ |
|  |  |  |
| ***Factor VII*** | Forward | 5´- GCTCCTGTGAGGACCAAATG-3´ |
|  | Reverse | 5´-CGCATGGGTAATCAACTGTC -3´ |
|  |  |  |
| ***Mat1a*** | Forward | 5´-GCCTTCATGTTCACATCAGA-3´ |
|  | Reverse | 5´-TGCACTCCTCCGTCTCGTCA-3´ |
|  |  |  |
| ***Slu-7*** | Forward | 5´-GAAGAAGGAGCTAGAAGAACAG-3´ |
|  | Reverse | 5´-CTTCCCATCGTAGTCAAACATCAG-3´ |
|  |  |  |
| ***Gapdh*** | Forward | 5´-GAATCCACTGGCGTCTTCAC-3´ |
|  | Reverse | 5´-CGTTGCTGACAATCTTGAGAGA-3´ |
|  |  |  |
|  |  |  |

**Supplementary Figure 1.** Gene expression of inflammatory cytokines (IL-6, IL-1b) in the CrLs in sham (grey bars) and 0.3 GBq group (green bars) when compared with controls (dashed line)


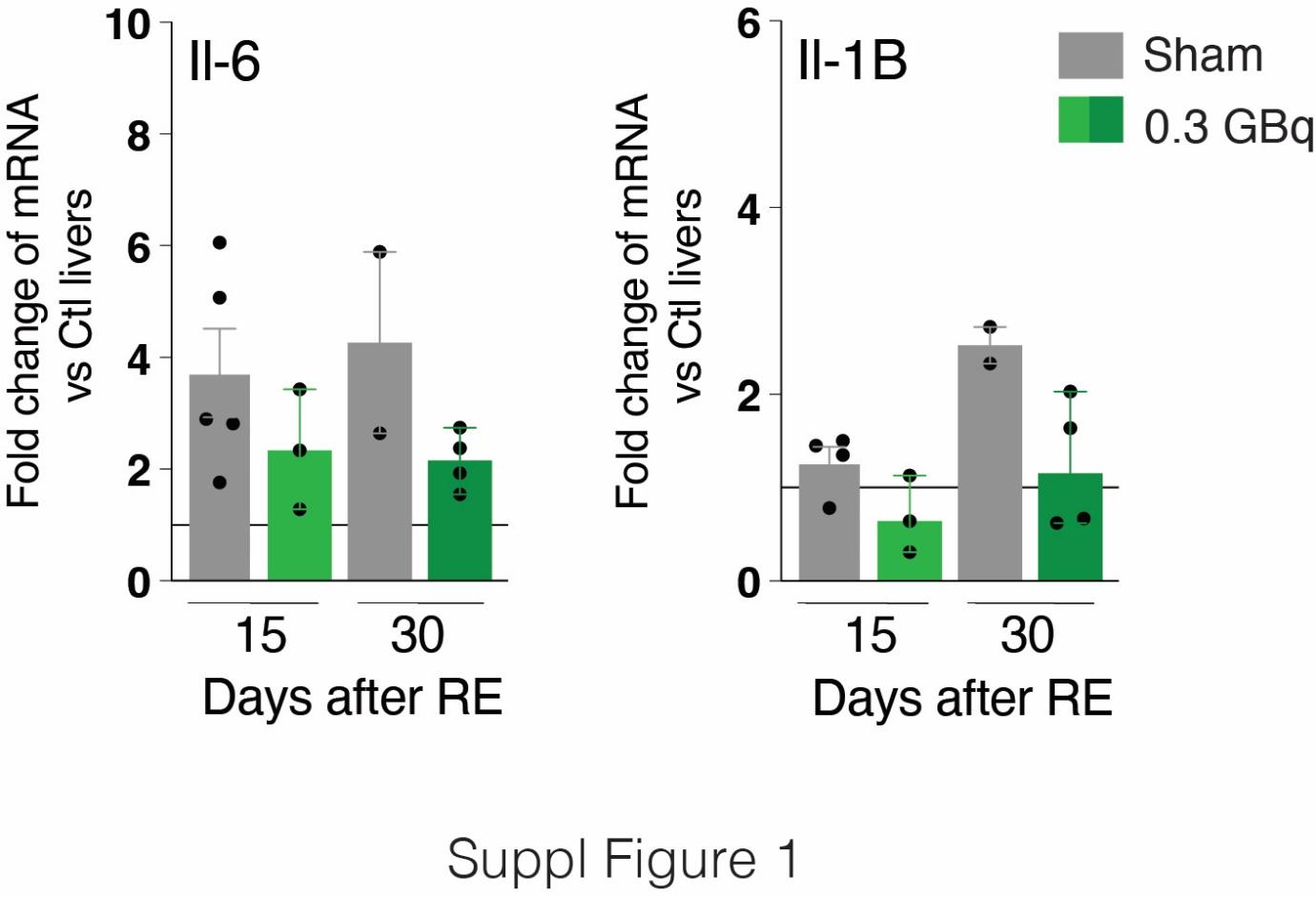
**Supplementary Figure 2:** Original films from two different exposure times of Western blot images showed in **Fig 7D**. Blots of phospho-STAT3, PCNA and B-Actin are shown. The left side of each film shows a 20 second-exposure. The mirrored image on the right side of each film is 1-minute exposure. The order of samples in the left side blots is as follows: Y8 (1.2 GBq, 15 days); Y4 (0.6 GBq, 30 days); Y14 and Y13 (0.6 GBq, 15 days); Y19 and Y16 (0.3 GBq, 30 days); Y12 and Y11 (0.3 GBq, 15 days); E2 and E1 (Sham group, 15 days); F9 and F6 (Fluor, 15 days), C3 and C2 (Control group, untreated). On the right side, the image is mirrored, so the order of the samples is reversed. Phosphorylation of STAT3 (Y705) did not show significant differences and was not included in the manuscript. On the other hand, the Y8 sample (the only surviving animal treated with 1.2 GBq) was not clearly seen in any of the blots and robust conclusions in 1.2 GBq-treated rabbits cannot be drawn.


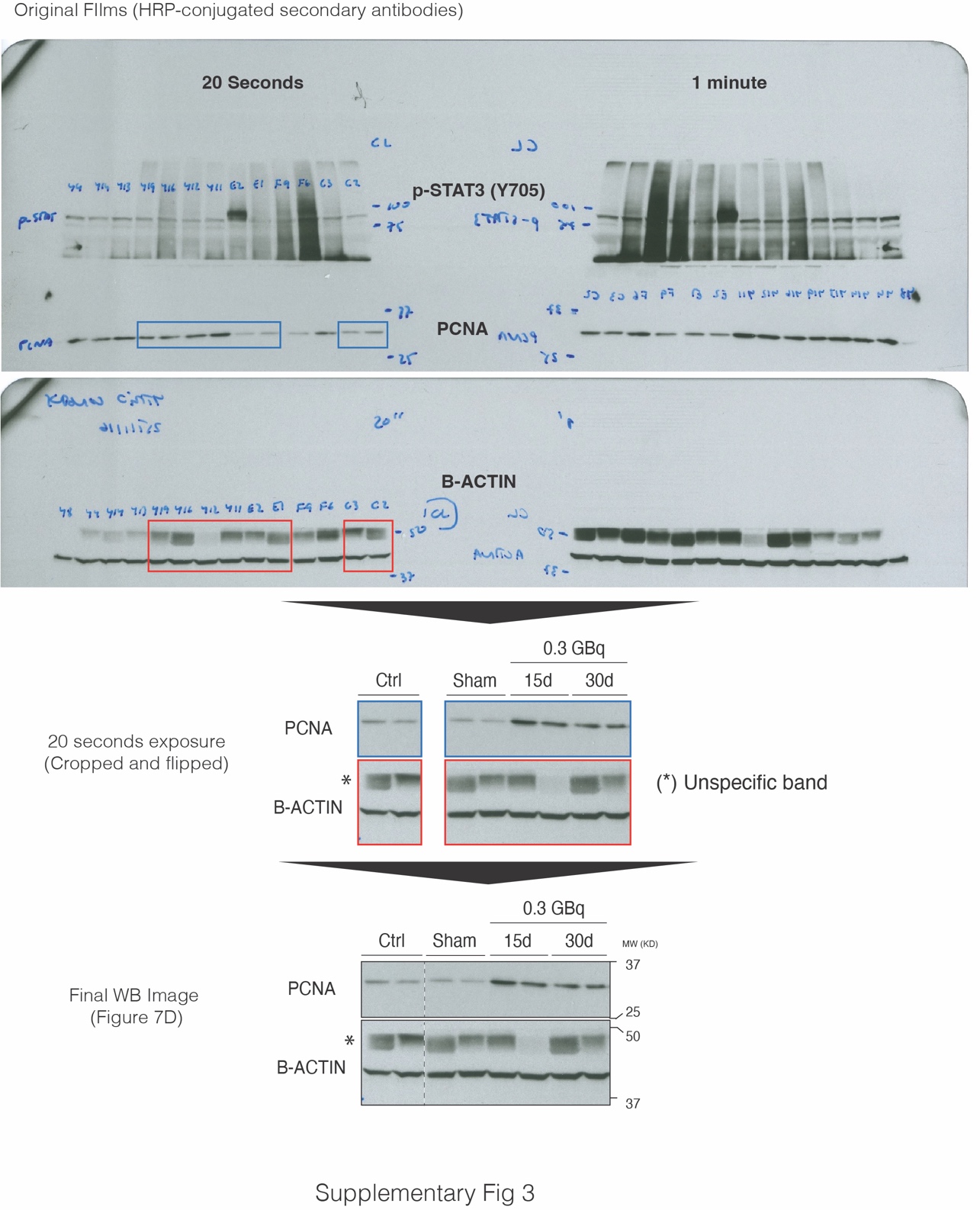


**Supplementary Figure 3: (A)** Scatterplot of CLV versus caudal liver weight (CLW) at sacrifice (Spearman’s correlation coefficient, r= 0.89). **(B)** Scatterplot of liver volumes at two measurements by same radiologist (Spearman’s correlation coefficient, r= 0.99).


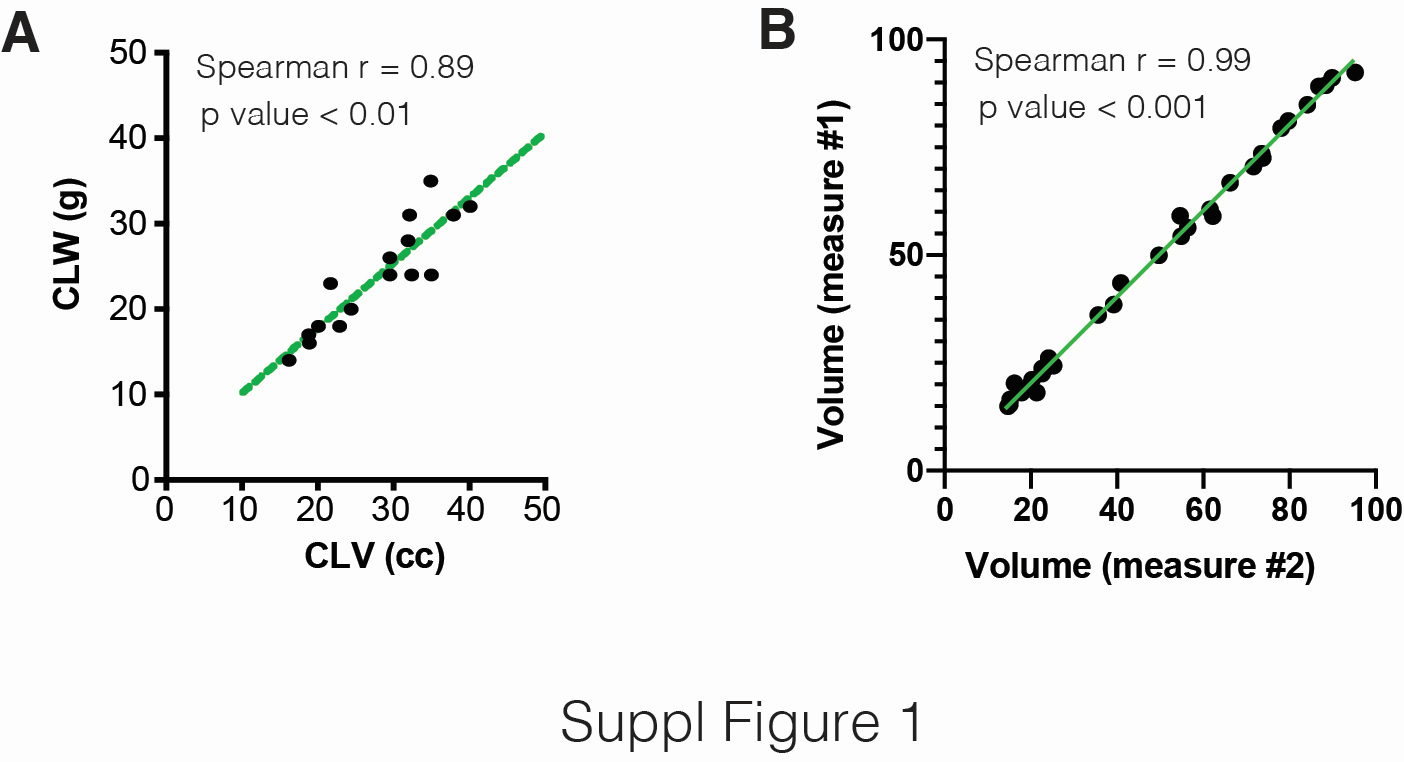

Supplement: Supplementary file 1 — Supplementary Information. [file 41598_2022_5672_MOESM1_ESM.docx]
